# Participant experiences in a decentralized clinical trial using digital health technologies: The ACTIV-6 study

**DOI:** 10.1017/cts.2025.69

**Published:** 2025-04-16

**Authors:** Carrie Reale, Janelle Faiman, Russ Beebe, Jessica S. Marlin, Sean Collins, David R. Boulware, Sarah E. Dunsmore, Florence Thicklin, Thomas G. Stewart, Adrian F. Hernandez, Russell L. Rothman, Christopher J. Lindsell, Shilo Anders, for the Accelerating COVID-19 Therapeutic Interventions and Vaccines (ACTIV)-6 Study Group and Investigators

**Affiliations:** 1 Center for Research and Innovation in Systems Safety (CRISS), Vanderbilt University Medical Center, Nashville, TN, USA; 2 Vanderbilt Institute for Clinical and Translational Research (VICTR), Vanderbilt University Medical Center, Nashville, TN, USA; 3 Department of Emergency Medicine, Vanderbilt University Medical Center and Veterans Affairs Tennessee Valley Healthcare System, Geriatric Research, Education and Clinical Center (GRECC), Nashville, TN, USA; 4 Department of Medicine, University of Minnesota Medical School, Minneapolis, MN, USA; 5 National Center for Advancing Translational Sciences, Bethesda, MD, USA; 6 Stakeholder Advisory Committee, Pittsburgh, PA, USA; 7 School of Data Science, University of Virginia, Charlottesville, VA, USA; 8 Duke Clinical Research Institute, Duke University School of Medicine, Durham, NC, USA; 9 Institute for Medicine and Public Health, Vanderbilt University Medical Center, Nashville, TN, USA

**Keywords:** Decentralized clinical trials, digital health technologies, research participant experience, recruitment, electronic data capture

## Abstract

**Background:**

Inadequate recruitment and retention impede clinical trial goals. Emerging decentralized clinical trials (DCTs) leveraging digital health technologies (DHTs) for remote recruitment and data collection aim to address barriers to participation in traditional trials. The ACTIV-6 trial is a DCT using DHTs, but participants’ experiences of such trials remain largely unknown. This study explored participants’ perspectives of the ACTIV-6 DCT that tested outpatient COVID-19 therapeutics.

**Methods:**

Participants in the ACTIV-6 study were recruited via email to share their day-to-day trial experiences during 1-hour virtual focus groups. Two human factors researchers guided group discussions through a semi-structured script that probed expectations and perceptions of study activities. Qualitative data analysis was conducted using a grounded theory approach with open coding to identify key themes.

**Results:**

Twenty-eight ACTIV-6 study participants aged 30+ years completed a virtual focus group including 1–4 participants each. Analysis yielded three major themes: perceptions of the DCT experience, study activity engagement, and trust. Participants perceived the use of remote DCT procedures supported by DHTs as an acceptable and efficient method of organizing and tracking study activities, communicating with study personnel, and managing study medications at home. Use of social media was effective in supporting geographically dispersed participant recruitment but also raised issues with trust and study legitimacy.

**Conclusions:**

While participants in this qualitative study viewed the DCT-with-DHT approach as reasonably efficient and engaging, they also identified challenges to address. Understanding facilitators and barriers to DCT participation and DHT interaction can help improve future research design.

## Introduction

Analysis of clinical trials data reveals a frustrating reality: Inadequate participant recruitment and retention remain a major barrier to achieving research goals [1–5]. Failure to adequately recruit and retain qualified participants leads to trial inefficiencies and disproportionate resource consumption, and it impairs researchers’ ability to draw conclusions. This may lead to ethical concerns about exposing participants to investigational therapies without corresponding gains in knowledge [1].

A critical but often overlooked component of minimizing participant burden is a lack of understanding of the reality of a participant’s day-to-day experiences in a clinical trial [4]. Prior research indicates participant perspectives are diverse and complex [6]. Reviews of clinical trial publications identified multiple participation barriers, including gaps in understanding among participants, overly complicated trial information, and frustrations with technology [6,7]. Reports of standardized methods for measuring participant experience are uncommon, but increased inclusion may be a crucial component of quality improvement in clinical trial research [8].

In research studies, digital health technologies (DHTs), such as smartphone- or tablet-based mobile apps, activity-tracking wearable devices, and telemedicine systems, that enable remote data collection and transmission from the participant’s location, have the potential to improve participant access and engagement while increasing research efficiency and effectiveness [9–11]. Participants in a variety of studies have successfully used remote DHT to complete clinical trial activities [12–14]. Catalyzed by the COVID-19 pandemic, a number of decentralized clinical trials (DCTs) employing DHTs have emerged in recent years [15–21]. DCTs preserve the rigorous randomized, controlled study design of traditional investigational site-based clinical trials but leverage DHTs to conduct many or all study activities at a distance without relying on in-person visits and data collection at a centralized research facility [15,16]. A more distributed study design offers potential advantages for improving the participant experience in clinical trial research by reducing participant burden [16,22]. DCTs employing DHTs also pose potential risks for participants, including data privacy and security concerns and increased technological learning curves to properly use DHTs [4,15]. Emerging regulatory and policy frameworks to support DCTs recognize the foundational role that DHTs play in remote trials and the importance of ensuring safe use [23].

Early evidence of participants’ experience in DCTs using DHTs is limited but promising. Daudelin et al. conducted a small qualitative study of patient and study personnel perspectives in a fully remote COVID-19 clinical trial [24]. The vast majority (96%) of patient respondents were more likely to participate in future studies if activities would not require in-person visits. Patient participants reported positive interactions with study personnel and the majority rated the remote technology (i.e., survey and telehealth platforms) as easy-to-use. However, data from the study personnel group indicated that technological barriers remained, since recommendations included usability improvements to the remote consenting software and ensuring participants knew how to use the technology.

Additional qualitative studies of trial personnel’s experience in DCTs with DHTs as well as publications disseminating results from the DCTs also offer potential insights into participants’ experience. Reports of modifications to patient-facing instruments, clarifications of study information based on participant inquiries, medication and equipment delivery issues, and protocol non-compliance (e.g., not taking medications) imply some participants may have struggled with survey question interpretation, managing remote trial instructions, and completing activities at home [21,25,26]. These findings suggest that while DCTs with DHTs are likely to improve important aspects of clinical trial participation and management, they also run the risk of shifting the burden of trial activities onto participants [26]. Further research into the remote trial participant experience is needed [9,27]. The goal of this qualitative study is to expand our understanding of participants’ perspectives in a large DCT leveraging DHTs.

## Materials and methods

After receipt of Institutional Review Board approval as an exempt study, we conducted focus groups with participants in a large, national DCT to systematically explore their experience enrolling, going through the consent process, and participating in trial activities.

### Clinical trial description

**Figure 1.**
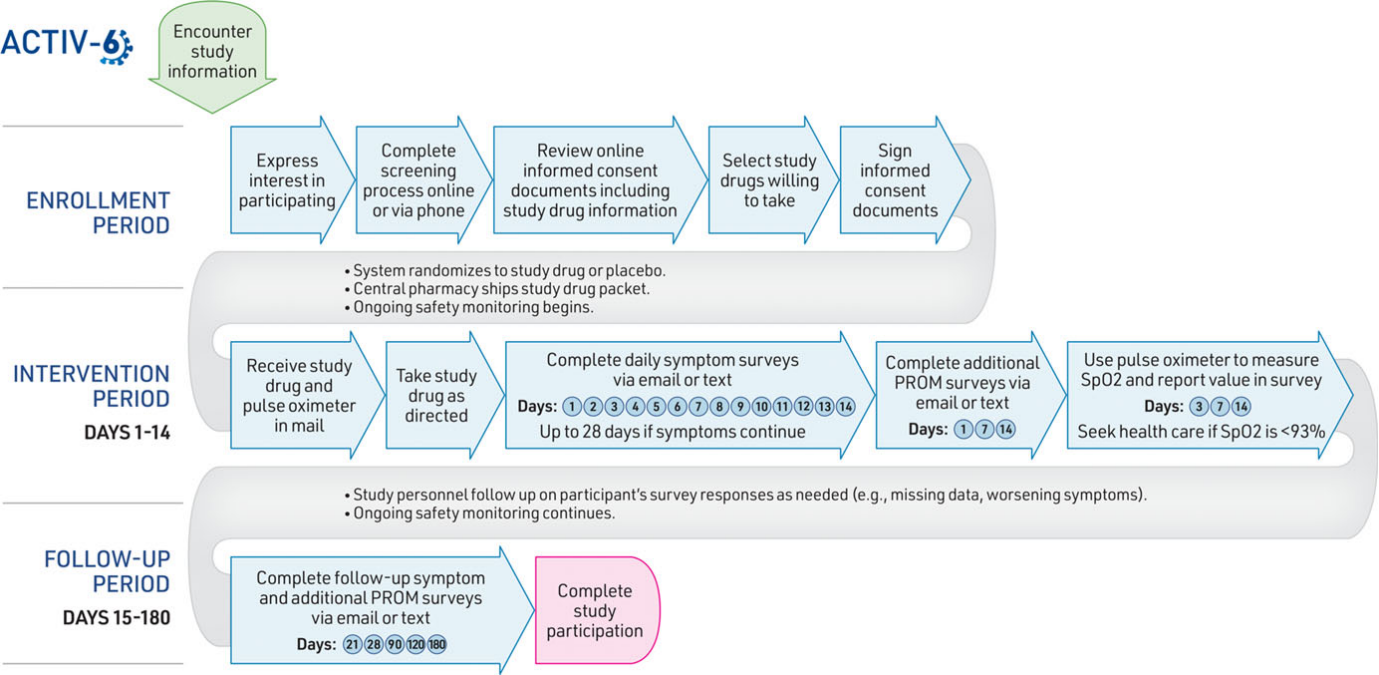
Participant activities in the ACTIV-6 decentralized clinical trial. Abbreviations: ACTIV-6, accelerating COVID-19 therapeutic interventions and vaccines (ACTIV)-6; PROM, patient-reported outcome measure.

The Accelerating COVID-19 Therapeutic Interventions and Vaccines (ACTIV)-6 Study is a large outpatient DCT designed to evaluate the effectiveness of multiple repurposed medications to improve recovery from mild-to-moderate COVID-19 in the outpatient setting [17]. Fig. 1 summarizes the activities a participant needs to complete when participating in the ACTIV-6 trial.

In the enrollment phase, interested participants are screened and enrolled through one of three pathways: 1) online through the ACTIV-6 web site, 2) a call center via a toll-free phone number, or 3) outreach through a local study site. Symptomatic adults aged 30 years or older with a confirmed positive SARS-CoV-2 infection are eligible to participate. Participants provide consent electronically and are screened for eligibility for the study drugs available at that time. During the consent process, participants indicate which available study drugs they are willing to take. The system then randomizes an individual participant to either placebo or one of the open study drug interventions for which they are eligible and willing to take. Participants receive the assigned study drug packet with instructions and a pulse oximeter, shipped overnight from a central pharmacy to their home.

During the 2-week intervention phase, participants take the study drug (or placebo) as directed and complete daily electronic surveys sent via email or text to report symptoms, healthcare utilization, and any questions or concerns. Surveys include questions from previously developed instruments and regulatory guidance on patient-reported outcome measures, such as the Columbia-Suicide Severity Rating Scale and FDA guidance on patient-reported outcomes in clinical trials [28,29]. The follow-up phase may last up to 180 days, with participants completing a series of electronic surveys at the designated intervals described in Fig. 1. If an individual chooses to stop taking their assigned study drug before finishing the indicated duration, they are asked to continue completing the remaining scheduled surveys. Study personnel perform ongoing remote monitoring for safety and efficacy throughout the intervention and follow-up periods. No in-person visits are required to complete study activities. Participants are compensated upon completion of study activities.

### Qualitative study protocol

For this qualitative study of the DCT experience, we conducted a series of small virtual focus groups with current and recently completed ACTIV-6 study participants.

### Recruitment

We sent recruitment emails to recent ACTIV-6 study participants as of January 2023 to elicit volunteers for the focus groups. We purposefully targeted individuals in either the intervention or follow-up study periods at the time of recruitment because we expected that these individuals would have a higher likelihood of recalling more details of their day-to-day DCT experience compared to individuals who had completed the trial months ago. Interested participants used an online reservation system implemented in REDCap [30], and a study team member followed up by email to confirm the date and time of the virtual focus group session.

### Focus group procedure

Focus group sessions lasted up to 60 minutes and were conducted remotely through a Health Insurance Portability and Accountability Act (HIPAA)-compliant version of Zoom videoconferencing software (Zoom Video Communications, San Jose, CA, USA). Two human factors researchers facilitated each virtual session, obtaining verbal assent from participants and providing session instructions, including ground rules designed to encourage respectful participation from all focus group members. For example, participants were encouraged to use Zoom’s interactive chat and gesture features (e.g., raise hand) to signal that they had something to add to the discussion if they felt uncomfortable jumping in verbally at any point in the conversation. Facilitators guided the group discussion through a semi-structured script probing different aspects of the participants’ experiences in the ACTIV-6 study and overall perceptions and expectations of the study. Specific topics explored included their motivation to participate, communication with study personnel, the enrollment process, study drug delivery, symptom surveys, and suggestions for improvement. Participants received a $35 electronic gift card by email after completing the focus group session.

### Data collection and analysis

All focus group sessions were audio recorded and transcribed for analysis. To protect anonymity and blinding within the ongoing clinical trial, we limited the demographic data collected from participants and did not link their focus group participation back to additional demographic data collected through the clinical trial. For reference, Table 1 provides the characteristics of the population sampled, all trial participants enrolled in the ACTIV-6 DCT during the focus group recruitment period (n = 1,060).

**Table 1.** Population characteristics of all ACTIV-6 participants enrolled in the DCT during the focus group recruitment period

Characteristic	*N* = 1,060
Age, median (IQR)	51.0 (40.8, 60.0)
Age Category	
Age<50	503 (47.5%)
Age≥50	557 (52.5%)
Sex	
Female	694 (65.5%)
Male	366 (34.5%)
Race	
American Indian or Alaska Native	16 (1.5%)
Asian	50 (4.7%)
Black, African American, or African	95 (9.0%)
Middle Eastern or North African	63 (5.9%)
Native Hawaiian or Other Pacific Islander	4 (0.4%)
White	774 (73.0%)
None of the above	62 (5.8%)
Prefer not to answer	24 (2.3%)
Ethnicity	
Hispanic/Latino	474 (44.7%)
Not Hispanic/Latino	586 (55.3%)
Region	
MW	187 (17.6%)
NE	160 (15.1%)
S	586 (55.3%)
W	127 (12.0%)
How did you learn about the ACTIV-6 study?	
Friend or family member	213 (21.2%)
My doctor or local health system	491 (48.8%)
Pharmacy or testing center	8 (0.8%)
Advertisement on the radio, television, or print	35 (3.5%)
A news story	5 (0.5%)
Internet and/or social media	255 (25.3%)
Unknown	53
Days between symptom onset and receipt of study drug, median (IQR)	5.0 (4.0, 7.0)
Days between symptom onset and enrollment, median (IQR)	3.0 (2.0, 5.0)
Unknown	1
Symptom burden on study day 1	
None	96 (9.7%)
Mild	526 (53.0%)
Moderate	364 (36.7%)
Severe	7 (0.7%)
Unknown	67
Symptom burden on study day 28*	
None	887 (86.5%)
Mild	119 (11.6%)
Moderate	17 (1.7%)
Severe	3 (0.3%)
Unknown	34

* Symptom burden on study day 28 values were imputed using the Last Observation Carried Forward method.

We used a grounded theory approach with open coding for our qualitative data analysis. Immediately following each session, the facilitators discussed concepts shared during the group conversation and made notes about potential ideas of interest to explore further during formal coding. The same two human factors researchers who facilitated the focus group sessions also performed the qualitative coding. The transcripts were imported into Dedoose (SocioCultural Research Consultants, Manhattan Beach, CA, USA) qualitative software for analysis. The coding process involved reading the transcript text, creating excerpts from the text, and assigning one or more relevant concept codes to each excerpt. The researchers met regularly to discuss code application, clarify code definitions, review proposed code modifications, and reach consensus if there was initial disagreement. The preliminary codes that emerged were then analyzed for repeated ideas and categorized by key concepts. The final set of agreed-upon coded excerpts were exported to Microsoft Excel (Microsoft Corporation, Redmond, WA, USA) for further thematic analysis and consolidation into the themes described below.

## Results

**Figure 2.**
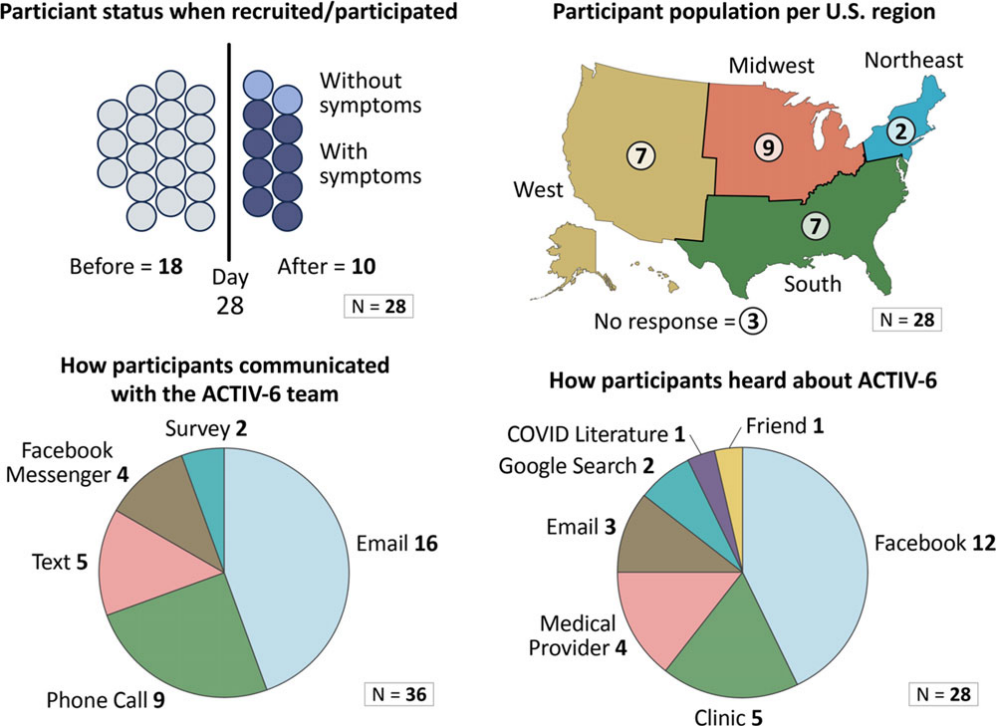
Focus group participant characteristics.

We sent recruitment emails to 200 ACTIV-6 participants, resulting in a convenience sample of 28 individuals aged 30 years or greater from different geographic regions of the U.S. who completed 1 of 12 focus group sessions in January–March 2023. Focus groups ranged in size from 1–4 participants. Fig. 2 presents the participant characteristics captured during the sessions. Our thematic analysis uncovered the following three major themes.

### Theme 1: perceptions of the experience of participating in a DCT with DHTs

The use of direct-to-participant procedures supported by technology was effective in achieving the basic study goals and moving participants efficiently through the scheduled study activities. Almost all participants reported a positive overall experience, even if minor issues arose during the study period (e.g., study drug delivery delays). None of the participants reported significant problems interacting with the study technology. In fact, gaining access to one of the technological components, the pulse oximeter, was cited by several participants as an unexpected benefit of participating. Having access to this tool for self-monitoring during their acute illness was reassuring.

“It was really simple, you know? They sent me the survey, I clicked right on it and filled out the information, and then it seemed like every day I’d just get a text in the morning. Then if I forgot, it would send me a reminder, which was good. So, yes. It was like a really foolproof way to make sure that they got the information across.” [Participant 39]

There was some variability in the logistical day-to-day experience among participants. The methods with which participants interacted with study personnel primarily depended on how they were enrolled in the study. Participants recruited through their existing healthcare provider reported a mixture of in-person (e.g., received information while at an office visit) and remote interactions, whereas those recruited through social media channels reported a fully remote study experience. Communication patterns also varied. All participants reported communicating with the study team via email and text, while some also spoke to the study team by phone one or more times. Despite this variability, participants reported that the different approaches were successful.

“…it was usually really quick response times, which again was really soothing as someone who is sick and on their own. Like if you just shoot out an email, it made it much more comfortable to take – you know, whether or not it’s a placebo. But it’s just knowing that someone’s going to promptly reply if you needed them to is very comforting.” [Participant 85]

Reminders to complete daily surveys and scheduled medication doses during the intervention period served as an important organizing framework for participants. In fact, some participants reported confusion and disappointment when the daily surveys stopped after 14 days (per the study protocol) and reached out to the study team to confirm that this was not an error.

“I was glad to get them every day and track my progress. So, it was really helpful.” [Participant 39]

“I actually called that number because I had stopped getting the surveys. So, they said it’s two weeks, and then they do, like, a month later, then two months later…So, I just have to wait for it.” [Participant 55]

Helping others by participating in research in general and more specifically improving the collective ability to treat COVID-19 were the most common reasons cited for participating. Having a loved one with severe COVID-19 or long COVID was another common motivation for participation. Several participants saw value in joining the study to ensure daily remote monitoring while sick and quarantined with COVID-19.

“I was also on my own when I had COVID, so it was also kind of nice just to have people to check in with me daily and a way to monitor my symptoms as a single person in a household just to watch myself, as well as try and do something helpful.” [Participant 85]

“I liked, you know, the little questionnaires that we would get every day. It helped me keep up with my symptoms. And to be honest, it’s probably not something I would have done if I wasn’t receiving, you know, the questionnaires, so that helped me.” [Participant 38]

Participants cited the ability to complete study activities from home on their own daily schedule as a highly appreciated aspect of the study design.

“…I live in a rural area in [state]…Even going to the grocery store is traveling, for me. But, yeah. It worked so much better from home because you can be relaxed and do your own thing, but still have to do the, you know, different things that they’ve asked in the study. And it only takes five, ten minutes, not even five minutes out of your day to do it.” [Participant 95]

### Theme 2: engagement with study activities

Focus group participants’ descriptions of their day-to-day study work suggested a high level of commitment to and engagement with the study activities during the intervention period among these individuals. Participants reported efforts to ensure that they fulfilled their study responsibilities, such as incorporating completion of the daily symptom survey into their morning online activities or adding the study drug dose to their normal medication routine, as well as efforts to make their data more meaningful to researchers, such as wanting to type comments in the survey to communicate symptom nuances to provide the study team with more context than the standardized questions allowed.

“…it would ask me about my pain, but it – my pain wasn’t always related to COVID. I think that’s what I wanted to say when I was taking those surveys because I had Lyme Disease this fall and I was having some residual pain in my knees, but I had that way before COVID.” [Participant 21]

“I have a lot of health problems, so I can’t, you know, when I’m normal I can’t go up and down stairs and, you know, do the other stuff that are on the list, too…it might have helped the information gatherers a little bit to know that I’m already disabled.” [Participant 16]

Managing study medication from home can be a complex process, but overall, participants coped well with the task. Some participants reported confusion about the dosage or duration of their study drug despite the instructions in the study packet. Most reached out to the study team to clarify these knowledge gaps. In one case, a misinterpretation of information in the daily surveys resulted in a participant electing not to take the study drug because their COVID symptoms resolved.

“When I had COVID, it was like a cold for, like, a day or two and it was done. So, I’m taking these surveys, daily surveys, and the questions are asking you if you’re taking the medication, yes or no. And there’s an answer in there that says you don’t have to if you’re feeling well. And no one ever said anything, so I haven’t taken the medication, but I’ve been feeling fine.” [Participant 55]

While patients were blinded to receiving active study drug or placebo, multiple participants conducted their own online research when the study drug arrived before taking it for the first time, looking up important safety information like indications, side effects, and potential drug-drug interactions of any potential active drug they might receive. In fact, one participant reported discovering a potential drug–drug interaction with their assigned drug through this personal research when they learned its pharmacological class and realized that they were taking another drug in this same category.

“I just knew that it was an FDA approved drug. And then, when it came, I looked it up. And I looked my medications up and saw that they didn’t mix.” [Participant 05]

This participant reported that they contacted the ACTIV-6 team, who confirmed this was a relative contraindication and supported them in their decision to not take the study drug. They also continued to complete the daily surveys despite not proceeding with the study drug component.

Participants generally found the symptom surveys efficient and effective, appreciating that the ACTIV-6 DHTs delivered a direct link via email or text reminder that allowed them to complete this task in a few minutes on the personal device of their choice (e.g., smartphone). However, participants reported uncertainty about how to correctly respond to some of the standardized survey questions. For example, many participants reported completing the surveys early in the morning when they received their first reminder but noted that several questions asked about their symptom severity “today.” Participants expressed uncertainty about whether to interpret this lookback period as “so far today” or “over the last 24 hours.” One participant noted their symptoms tended to fluctuate over the course of the day, starting out mild in the morning and typically worsening in the evening. This made it difficult to assign a single severity rating for “today.”

Other participants were unsure how to interpret the symptom questions about general pain when living with chronic pain (e.g., to include their baseline pain or only new pain that they thought was from COVID-19) and physical activity when comorbidities limit mobility (e.g., not being able to walk up the stairs even when not sick from COVID-19). Some participants also struggled with questions about daily activities while under quarantine, such as running errands or engaging in social interactions, as they were concerned that the researchers might interpret this as resulting from physical illness rather than public health policy. Participants described a desire to provide the researchers with the most useful information possible when completing the surveys.

“…because I have osteoarthritis, I would have – was [I] in pain due to COVID? It hurt when I coughed, I had a sore throat, where were they going with this question? Maybe this could have been, you know, the same thing, due to COVID, or maybe even a little box that I could have typed in, you know, my back is hurting, my knees are hurting, something like that.” [Participant 51]

Participants described other gaps in knowledge about the study protocol and upcoming activities they would be responsible for, including duration of the follow up period, timing of surveys after the intervention period, and compensation timing and amount.

### Theme 3: Trust

A few participants described feeling doubts about the legitimacy of the study when encountering recruitment materials online. This initial lack of trust was associated with recruitment and enrollment through social media platforms; participants who first learned about the study through their healthcare provider or other representative of a known healthcare organization did not express these concerns. This uncertainty caused some to question whether they should volunteer to participate.

“I was a little bit suspicious as to whether I was joining something that was legitimate or not, because there’s so many things out there. And since I had heard of [academic medical center] before and since there was a [academic medical center] link to where I found the information, I wasn’t quite as worried. But I could have had a little bit more reassurance from the start…Having a little bit more clarity about how that was all attached together in the beginning would have made me feel more comfortable, knowing that I had joined something that was legitimate. Since I was getting drugs and everything from them, I wanted to make sure that it was safe.” [Participant 26]

However, one participant shared that encountering negative comments on social media regarding the study actually motivated them to seek more information.

“…I was curious because people in the comments were poo-pooing it, so I wanted to see what it was about…I’m assuming they were COVID deniers.” [Participant 21]

While the majority of participants appreciated the streamlined nature of electronic communications supported by study DHTs, the lack of face-to-face or interpersonal connection with study team members may have contributed to decreased trust in the overall study for some participants.

“The initial string of messages was fine. I felt like I knew I was talking to a real person, and they were responding to me, but after that it was all, like, the emails. There was no personal contact with anybody. It was just all automated.” [Participant 21]

## Discussion

The findings from our qualitative study of participants’ day-to-day experiences in a DCT with DHTs indicate that participants may view this research approach as effective, efficient, and engaging. The thematic analysis provides the ACTIV-6 study team and other researchers conducting DCTs using DHTs with insights to potentially enhance study materials, processes, training, and technology. Understanding the facilitators and barriers to participating in DCTs and interacting with DHTs in a research context can help inform future research design more broadly. This knowledge can also contribute to the development of improved strategies to overcome research mistrust and increase recruitment effectiveness by characterizing participant perspectives in these crucial areas of research.

Our findings align with the results of early qualitative studies of patient and study personnel experiences in DCTs, strengthening the evidence for the ability to participate remotely at a convenient time of day through DHT and virtual symptom monitoring while sick at home as important facilitators of participation [21,24,26]. We discovered similar barriers as well, including difficulty interpreting survey questions and confusion about study activity timelines [21,24]. In another COVID-19 DCT, Avula et al. reported issues with participants not taking study drugs, but did not have data on what led participants to make these decisions [25]. The rich examples of medication-related behaviors uncovered in our study shed more light on potential contributing factors to the complex issue of poor adherence in a remote context, including misinterpretation of trial instructions, and self-identified relative contraindications.

Many of the barriers that participants identified during our focus group discussions could be addressed. For example, digital surveys could incorporate real-time, on-demand guidance for common interpretation questions as participants complete a task to help them provide the best quality data possible. Variability in survey question interpretation is an ongoing challenge in patient-reported outcome research [31]. Incorporating questions from more than one previously validated instrument may result in participant surveys with wording variance and changing or unstated lookback periods, increasing the likelihood that participants will have questions about how to accurately respond. It may be more advantageous to move away from the strict use of “validated” survey questions as originally worded and instead collaborate with participants to craft questions establishing the right language for the specific study setting. Application of human-centered design principles [32] to DHTs and DCTs could similarly enhance the participant experience for other study tasks, such as medication adherence and proper use of clinical devices (e.g., pulse oximeters).

Participant engagement with and knowledge of study activities are critical factors for DCT success. ACTIV-6 has a dedicated study website (www.activ6study.org) that includes recruitment information and a section where participants can report concerns. To promote engagement without face-to-face interactions and address the other knowledge gaps, we identified regarding study schedules and protocols, medication adherence, and timing of future activities, DHTs could be further leveraged in DCT design through the expansion of such informational websites. More robust online tools could incorporate a secure participant portal to provide a centralized resource for individualized trial information to better support the participant journey and create opportunities for meaningful participant interactions, such as through the collection of ongoing feedback on the participant experience. Following a human-centered approach to the design of such a system by involving participants in the design and development of the tool will help ensure it meets their needs [26,27,33].

A substantial number of our focus group participants learned about ACTIV-6 through social media. Engaging with social media for DCT recruitment may have unexpected consequences that are both positive and negative [34–36]. On the positive side, we found that participants generally responded well to interacting with researchers on social media platforms that they were already engaged with online. This interaction successfully supported recruitment and enrollment for geographically dispersed participants. However, participants’ concerns about the legitimacy and safety of a study presented to them solely through social media need to be further addressed. Similar to the findings of Pullen et al. in another COVID-19 DCT, people who learned about ACTIV-6 through social media had to decide to participate in the face of potential misinformation and negative remarks via social media [21]. Investigators should carefully consider the potential downsides to social media recruitment and develop mitigation strategies before implementation, especially if the study addresses a potentially divisive subject. Additional research is needed to identify strategies to leverage these dynamic online spaces without fueling mistrust and misinformation about DCTs.

This study has important limitations. The use of a convenience sample of self-selected participants limits the generalizability of our findings to the entire ACTIV-6 participant population as well as to the populations of other DCTs. For example, it is possible that participants with higher levels of engagement with a clinical trial or greater comfort with technology were more likely to volunteer for additional study activities like our virtual focus groups. To decrease risk to participants’ privacy in the clinical trial, we did not collect detailed demographic data from the focus group participants during the group sessions nor were we able to link focus group participants back to their clinical trial study demographic data. The focus groups captured participant perspectives at only one point in time during the DCT.

Future studies could further expand our understanding of participant experiences in DCTs by incorporating more opportunities for participants to provide feedback throughout their clinical trial participation. Qualitative aims measuring participant experience and satisfaction could be included in overall DCT research plans, such as scheduled participant experience checkpoints after completing key study activities. Importantly, additional research is needed to explore the experiences of DCT participants who may be less engaged than many of our focus group participants appeared to be. Understanding what leads some participants to miss data collection or other study activities, disengage, or drop out entirely once enrolled is critical to improving DCT participation, retention, and outcomes.

This study advanced our understanding of participant experiences in DCTs using DHTs. Better knowledge of participant experiences in DCTs and barriers to participation can help inform the development of strategies to overcome research mistrust and increase recruitment effectiveness, engagement, retention, and data quality.
